# Effects of One-a-Day Foot Patrols on Hot Spots of Serious Violence and Crime Harm: a Randomised Crossover Trial

**DOI:** 10.1007/s41887-021-00067-2

**Published:** 2021-09-06

**Authors:** Lewis Basford, Chris Sims, Iain Agar, Vincent Harinam, Heather Strang

**Affiliations:** 1 Essex Police, Essex, UK; 2 University of Cambridge, Cambridge, UK

**Keywords:** Foot patrol, Hot spots, Residual deterrence, Crime harm index, Randomised experiment, Street-visible crime, Crossover design

## Abstract

**Research Question:**

Does one foot patrol per day (15–20 min) conducted in serious violence harm spots reduce street-visible crime harm and frequency relative to no foot patrol in the same hot spots, and if so by how much?

**Data:**

We identified 20 hot spots of 150m^2^ each on the basis of community violence defined as serious assaults, robbery, and drug dealing in the Southend-on-Sea area of Essex Police, with boundaries geo-fenced to collect GPS measures of foot patrol presence generated by hand-held electronic trackers issued to officers directed to perform patrols. All street-visible crimes were counted for each of the 90 days of the experiment in each hot spot.

**Methods:**

Daily random assignment of each hot spot to either control or treatment conditions (*N* = 90 X 20 = 1800 place-days) prescribed 720 place-days to receive extra patrols by Operational Support Group officers, which were compared to 1080 place-days with no extra patrols, using an intent-to-treat design, with 98% compliance with assigned treatments. Independent measures of other police presence in the area were tracked by the force-wide GPS telematics measures. All crimes were coded with the Cambridge Crime Harm Index for their CHI value.

**Findings:**

The 20 harm spots comprised just 2.6% of the geographical area of the Southend-on-Sea area, with 41% of all its Cambridge CHI crime harm in the year preceding the experiment. Background patrol presence was about 2 min per day on control days and 1 min per treatment day. Crime harm scores for serious community violence were 88.5% lower on experimental days with extra patrols (mean = 1.07 CHI per treatment place-day) than without it (mean = 9.30 CHI per control place-day). Crime harm scores for all street-visible offences were 35.6% lower on treatment days (mean = 7.94 CHI per treatment place-day) than control days (mean = 12.33 CHI per control place-day), while the mean count of all street-visible offences was 31% lower on treatment days (mean count = 0.09 crimes per treatment place-day) than on control days (mean count = 0.13 crimes per control place-day). The estimated effect of the 720 days with 15-min patrols was to prevent crimes with recommended imprisonment of 3161 days, or 8.66 years.

**Conclusion:**

The use of two-officer foot patrol can be highly effective at preventing serious violence in street-visible settings in small areas in which such violence is heavily concentrated.

## Introduction

This study reports on a practitioner-led randomised controlled trial of one-a-day foot patrols in Southend-on-Sea, Essex, between 20th July 2020 and 17th October 2020. The study sought to test high visibility foot patrol on street-based violence within the most harm-generating locations. The 20 highest harm spots of 150m^2^ were selected for intervention. The harm spots represented just 2.6% of Southend’s geographical area but contributed disproportionately to crime harm for community violence over the preceding 12 months, with 41% of all Cambridge Crime Harm Index (Sherman, Neyroud & Neyroud, 2016) harm for offences in the 12-month period leading to March 2020.

Officers from the Essex Police Operational Support Group were tasked to drive to the designated harm spots, park their police car in a highly visible location, and undertake foot patrol for 15 min. All officers were subject to a briefing that covered the research literature on hot spots policing, different types of intervention, and reasons that a community-based approach had been chosen for this study. Officers were also briefed on the electronic tracker’s capability to plot their whereabouts every 5 s. Daily feedback was then provided on the previous day’s visits and crime levels.

During two-officer foot patrols within the harm spots, officers were directed to undertake a minimum of 15 min and a maximum of 20 min of foot patrol with the police vehicle parked in a prominent and visible position. Officers were further guided to engage with the public in conversation and adopt a public engagement approach rather than seeking enforcement opportunities such as stop and account or stop and search (unless presented with spontaneous incidents or information that required police intervention).

### Background: Hot Spots Policing

The concept of hot spots policing began with the observation that 3% of places in Minneapolis were responsible for 50% of all calls for service (Sherman et al., 1989). Other studies since then have found similar observations and similar concentrations of crimes in micro-locations, especially for serious violence. Essex Police recently observed that 37% of all knife-enabled offences in the last 3 years took place in just 0.4% of all 200 m street segments and 60% of all community violence took place within 0.8% of all 200 m street segments (Essex Police, 2020).

These concentrations of high harm in a tiny fraction of all places invited experiments in concentrating police presence in the same fraction (e.g. Sherman & Weisburd, 1995). Systematic reviews of over 40 experiments have identified the crime control benefits of hot spots policing from 1989 to 2017 (Braga et al., 2014, 2019). Yet widespread implementation remains stagnant in most police forces across England and Wales. In her address to the Police Foundation in 2015 by then-Chair of the National Police Chiefs Council, Chief Constable Sara Thornton, she stated, “Hot spot policing is one of the strongest examples of using research to improve policing and, therefore, being able to demonstrate effectiveness” (National Police Chiefs Council, 2016). Yet even in 2021, we do not see wholesale implementation of this idea in any UK police force.

### What Should Police Be Doing at Crime Hot Spots to Reduce Violent Crime?

Braga et al. (2014, 2019) observed a mounting mass of research signifying an evidence base for directed police patrol and proactive and targeted arrests as well as a problem-orientated policing (POP) approach. One example is the Braga et al.’s (1999) randomised controlled experiment with the Jersey City Police Department to evaluate problem-oriented policing (POP) approaches through the allocation of twenty-four high crime intersections, split into twelve pairs of similar crime output, with one intersection of each pair receiving treatment and the other control conditions. The POP saw all 12 treatment locations receive aggressive order maintenance policing, supplemented with other interventions such as drug enforcement, cleaning of shop fronts, street cleaning, enhanced lighting, and eviction of troubling tenants. In total, there were 28 different treatments used in the 12 treatment areas. The varying use of all 28 treatments were analysed in the study, but it could not show which of the treatments had the optimal impact. Analysed results revealed a significant reduction at treatment locations against control for both total crime count, which varied throughout all six crime indicators (including robbery, assaults, and narcotics) as well as calls for service (street fight, nuisance/disorder, drugs [Braga et al., 1999]).

In the summer of 2009, in Philadelphia, 200 police officers across 60 violent hotspots implemented a randomised controlled trial to test the effectiveness of foot patrol against violent crime. The experiment found substantial reductions in crime, with treatment locations reducing counts of violent crimes compared to control locations by 23% (Ratcliffe et al., 2011).

## Residual Deterrence

While foot patrol was the exclusive treatment in the Philadelphia trial, two recent and supportive studies suggest the additional benefit of “residual deterrence” (Barnes et al., 2020; Ariel et al., 2020) and the temporal “free bonus” (Sherman, 1990) period of sustained deterrence after the policing activity has ceased. The Barnes et al.’s (2020) “so what” question in their Western Australia study was “how minimal can minimalist patrol be?” What they were asking is how minimal can patrol be to get optimum residual deterrence. Their experiment produced a 41.6% reduction in crime harm through an average of 13.8 min of patrol per patrol day, in a “crossover” design comparing the same hot spots on days with and without short patrols (on bikes, on foot, or in cars—any kind of patrols. The experiment by Ariel et al. (2020) in the London Underground showed that 2-officer foot patrols on higher-crime Tube station trackside platforms reduced crime all over the station, including at the level of the turnstiles many feet up from the platforms. They also reduced crime on days when there was no patrol, relative to a fixed control group that never received these patrols.

One of the best-known discoveries was Koper’s (1995) observational study of data from the Minneapolis preventative patrol study (Sherman & Weisburd, 1995), which found the optimal patrol length for residual deterrence to be 15 min—with diminishing returns on investment at greater lengths of patrol. Koper (1995), and more recently, Ariel et al. (2020) also demonstrated that the main element of deterrence occurred post-patrol and when police were not present. Ariel et al. (2020) showed that 97% of the total effect of foot patrols on crime prevention in the London Underground study occurred when police were not present, but in places where they did patrol regularly (and unpredictably).

The Barnes et al. (2020) study also showed a distinct “cliff edge” of residual deterrence with noticeable increases in crime after 4 days of no patrol and after an average of just 13.8 min of presence preceding those no-patrol days. This finding underlines the need for further studies in today’s environment of increased implementation of hot spots policing.

### Crime Count vs Crime Harm

The use of a crime harm index is essential to policing experiments on reducing street-visible violence. The recognition that all crimes are not created equal, and of the need for a Crime Harm Index (Sherman, 2013), has seen sustained progress towards organisational adoption in recent years. Specific indexes have been created in recent years that encompass harm relative to all crime, including a court records approach (Francis et al., 2005), an assessment of harm framework (Greenfield & Paoli, 2013), a sentencing gravity score (Ratcliffe, 2015), and a crime victim survey score (Ignatans & Pease, 2015). It is the Cambridge Crime Harm Index (CCHI), however, (Sherman, Neyroud & Neyroud, 2016) that has evolved and developed in policing organisations throughout the UK and across the world (Mitchell, 2017; Braga et al., 2014, 2019; House & Neyroud, 2018; Karrholm et al., 2020).

Reasons for a more consistent adoption of the Cambridge Crime Harm Index include its simplicity of application, consistent metrics of sentencing start points, and cost-neutral application within an organisation (Sherman et al., 2016). The Cambridge Crime Harm Index consistent metric is taken from sentencing guidelines and the starting point of the total days in prison a first-time offender convicted of that offence would receive. The starting point removes other uncontrollable metrics such as how many previous offences a suspect had previously been convicted of, first point guilty pleas, or other mitigating circumstances presented to the court, which in real-life application would see each person convicted of the same crime receiving varying sentence lengths (Sherman, et al. 2020).

Although significant progression has been made in the quest to present clarity of the data recorded by police and a true reflection of public safety, there remains a reluctance to move away from traditional crime counts and raw numbers. Sherman et al. (2020) therefore proposed further precision in counting crime through seven statistical series for counting crime usefully while remaining informative to the public and more useful for police decision making.

At present, the UK is utilising multiple methods with crime count remaining prevalent but complemented by the Cambridge Crime Harm Index (Sherman et al., 2016) and the Office of National Statistics (ONS) “Severity Score” index (ONS, 2020). The ONS Severity Score is seen as a backward-facing assessment with the average sentences for offences calculated from the previous 5 years data held by the Ministry of Justice. Although sentences would have been adjudicated through the judiciary with previous offending, mitigating, and aggravating factors being presented, Sherman et al. (2016) suggested, and the ONS (2020) remarks in its limitations, that the ONS approach is open to the current “judicial and public mood” with recent cases seeing a move towards stronger sentences for assaults against emergency workers, knife crimes, and noxious substance assaults. When considering its implementation and associated cost to the public purse, the ONS Severity Score requires continued investment to capture and collate the data, compared to the cost neutral application of the CCHI.

## Research Question

Does one foot patrol per day (of 15 to 20 min) conducted in serious violence harm spots reduce street-visible crime harm and frequency relative to no foot patrol in the same hot spots, and if so by how much?

## Data

We identified 20 hot spots of 150m^2^ each on the basis of community violence defined as serious assaults, robbery, and drug dealing in the Southend-on-Sea area of Essex Police, with boundaries geo-fenced to collect GPS measures of foot patrol presence generated by hand-held electronic trackers issued to officers directed to perform patrols. All street-visible crimes were counted for each of the 90 days of the experiment in each hot spot.

## Methods

### Setting

Southend-on-Sea is a coastal town with a population of 183,100 (89,100 males, 93,300 females), which doubles with daily visitors during the months of June, July, and August. Additional social data provided by Southend-on-Sea District Council reveals that there are 5700 households within the district that contain occupants who are all unemployed, which equates to 10.6% of all households against 14.6% nationally.

### Sample Selection: Targeting Harm Spots of Community Violence

The identification and selection of crime harm spots was performed in four steps. First, a crime query for violence and associated crimes led to the following section criteria in the ATHENA crime records system. First, the categories of violence with injury, robbery, and drug trafficking were the “community violence” crimes used to select the areas of concentration. Domestic abuse offences were excluded because they generally occur indoors out of the sight of police patrols. Second, the data analysis period was the 12 months to 23rd March 2020, the official date of the COVID-19 lockdown in the UK. An ATHENA enquiry indicated that there were 1381 offences in total for Southend-on-Sea contributing to a cumulative CHI harm total of 283,546 years of recommended imprisonment. The breakdown is displayed in Table 1 below:

**Table 1:** Southend-On-Sea community violence crime count and crime harm 2019–2020

Crime	Sub	Count	Harm
VWI	GBH/Att murder	111	165,710
VWI	Other wounding	967	22,151
Robbery	Personal	186	67,890
Robbery	Business	17	6205
Drug trafficking	PWITS	100	21,590.5
		1381	283,546

Third, a KDE (kernel density estimation) hotspot technique was used to identify clusters of crime within a 150-m area, weighted by the crime harm index to create harm spots. In Southend-on-Sea, most hotspots of violence were highly clustered or around “hot points” rather than being dispersed. Violence in Southend-on-Sea is intensely clustered in the central business district/town centre areas.

**Fig. 1:**
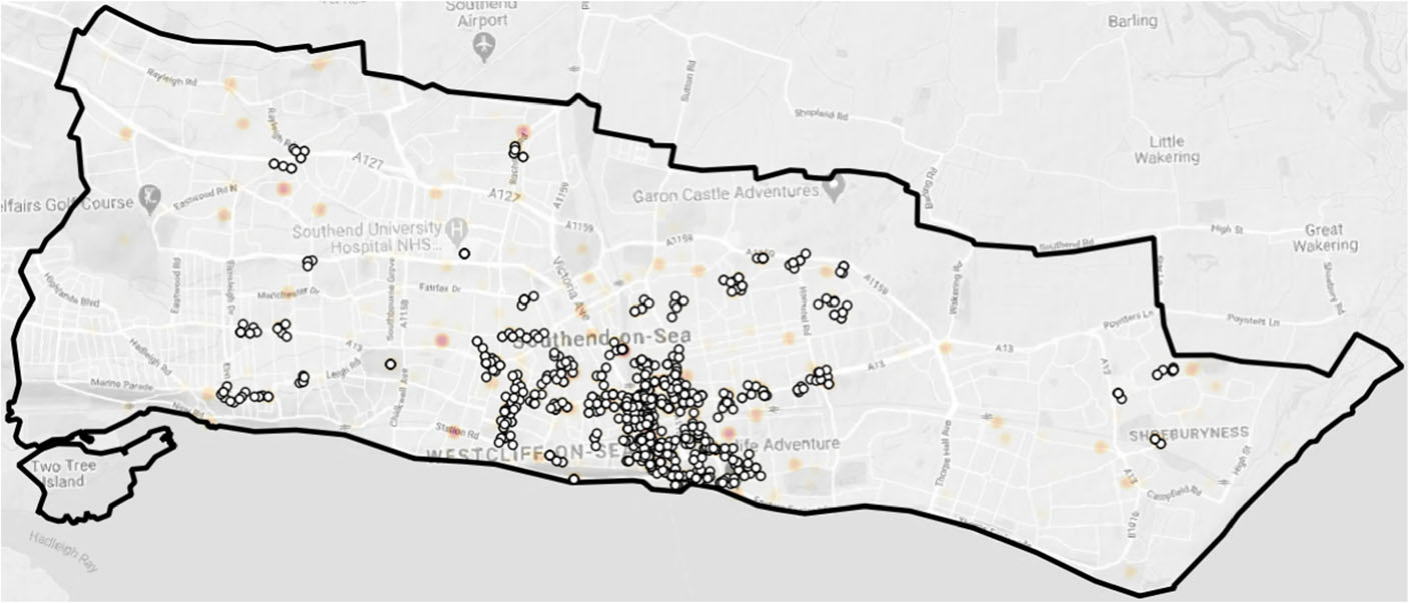
Violent crime clusters with 4 or more offences within 100 m

Fourth, an additional test was undertaken to identify which harm spots were created by extremely low frequency of very high harm events, which typically would be a geographically isolated assault with intent to cause serious harm (1* Grievous Bodily Harm). Any harm spot clusters with less than 4 eligible crimes, equivalent to less than 1 offence every 12 weeks, were omitted. Figure 1 illustrates several harm spots where there is no clustering of violence offences within 100 m. Two of the main harm spots that were excluded are Southend University Hospital and Southend Police Station as we already knew that geo-coding errors generated clusters of crimes for these specific locations.

**Fig. 2:**
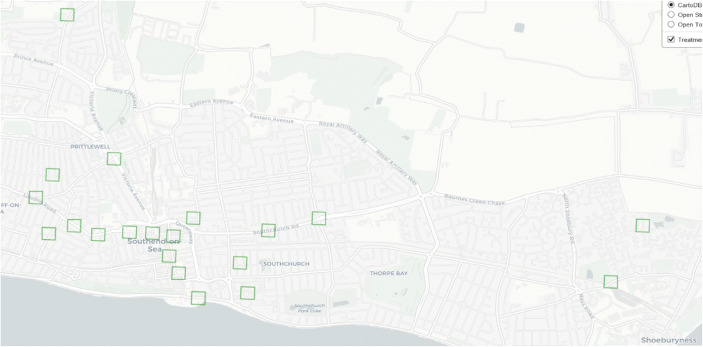
The harm spots in Southend-On-Sea

This method included the rule that where harm spots clustered, artificial grids were created based on centroids (coordinates) at the centre of the harm spots. The top 20 harm grids were selected for targeting as both control and treatment sites utilising the crossover design, with each one roughly 150 m in size with a 100-m buffer (Fig. 2).

Within these grids, 20% of all violence and 41% of all harm were occurring in 2.6% of the geographic area for a total of 277 offences in 2019–2020.

### Experimental Design

The randomised allocation was completed using a pre-designed randomiser in Excel and set on day one of the experiment with no alterations during the 90-day period.

Over the 90-day study, each harm spot was defined by control or treatment through randomisation and analysed on the treatment based on its intended delivery, ensuring the studies review is based on the intention to treat (ITT) analysis. This ensures that any place-days that a treatment harm spot was not attended, it would remain, for purposes of analysis, within the treatment group for that day—and vice versa with place-days that were assigned to no-patrol control status but went on to receive patrol treatment in error.

### Implementation

The eight harm spot allocations for each day were printed to give officers the location of only those harm spots requiring a visit for that day. The removal of other harm spots from the prints was to ensure a greater level of compliance. These prints were secured in a daily pack which was sealed at the point of randomisation. The packs were pre-printed each week and labelled by date with officers locating the pack and commencing patrol.

Officers were pre-allocated the overtime space to undertake the treatment. As such prior to the deployment, each officer was emailed the briefing as well as having a printed version within the pack. The briefing provided the officer with all the relevant details on what harm spots are and the theory behind the 15 min patrol period and incorporating the proposed benefits. The briefing also provided a template for officer returns and how to utilise the Global Positioning System (GPS) Tracker.

For this experiment, a “mission” was uploaded, for all 20 harm spots with scene photography and a geo-fenced area, onto the handheld device system. That system then sent an alert sound to the handheld device that officers were issued with to tell them that they were within the harm spot. Officers further developed this system during the deployment by adding markers for the optimum places to park the vehicle and any other location information that they felt may aid other officers attending the harm spot on a future treatment day. All officers had access to the software a backup aid was produced for officers to utilise, which was an application called “What 3 Words.” That geographical locator software was pre-loaded within an Essex Police mobile handset or available privately on personal phones. This allowed the officers to confirm that they were always in the correct location.

Prior to the start of the trial, the first author briefed departments that would have direct impact on the trial such as the Force Control Room and Operational Support Group Commanders to ensure they understood the strict requirements around compliance and ring-fenced patrol status they were provided for the visits. This meant that a layer of authority on not removing the resource for anything other than immediate threat to life incidents was considered during deployments. Each department was then given a 15-min briefing by the first author, in conjunction with the lead analyst (third author), to provide a complete overview of the project and to answer questions from officers, such as “so what?” This briefing was also provided, in separate sessions, to communication officers on dispatch, control room inspectors, and local field supervisors within Southend. These efforts attempted to foster a better understanding of the rationale for the hot spots patrol resource being protected for the deployment period.

#### Tracking

Tracking to measure whether officers were patrolling where they were deployed was monitored in three ways: GPS trackers with geo fence mapping panel notifications, the “telematics” system, and officer’s written reports.

The body-worn GPS trackers given to each patrol pair had a dedicated mapping panel for recording location data every 5 s. Predesigned geo-fenced harm spots were pre-loaded to track entry and exit times as well as speed of the tracker so that dip sampling of foot patrol could be undertaken.

The GPS trackers are small and compact at 70 × 39 × 20 mm and weigh only 65 g making them compact enough to add to the police equipment holsters. With up to 15 days battery life from a 2600 mAh battery, the reliability for the periods covered by the officers guaranteed significant tolerance for capturing the treatment period. The GPS tracker utilises a multi-roaming SIM card connected to the best cell network at any given location, and recording data locations every 5 s on the mapping panel. Providing real-time management and oversight throughout the experiment, automated emails for entry and exit of the harm spots were sent to a dedicated email account which prompted a real-time monitoring of the treatment time that was being delivered.

### Measuring Other Patrol Presence: Telematics

To measure the potential interference of treatment from other officers not working within the trial, a daily report from telematics (GPS data from all police vehicles) had been set up with its own geo-fenced area of all 20 harm spots. The telematics system provided a report on any police vehicle that remained within the harm spot for more than 90 s. This report allowed for the discounting of vehicles on random patrol or passing through on emergency calls. Those vehicles within the harm spot for over 90 s were then manually reviewed and reported within the analysis as additional treatment/dosage.

**Fig. 3:**
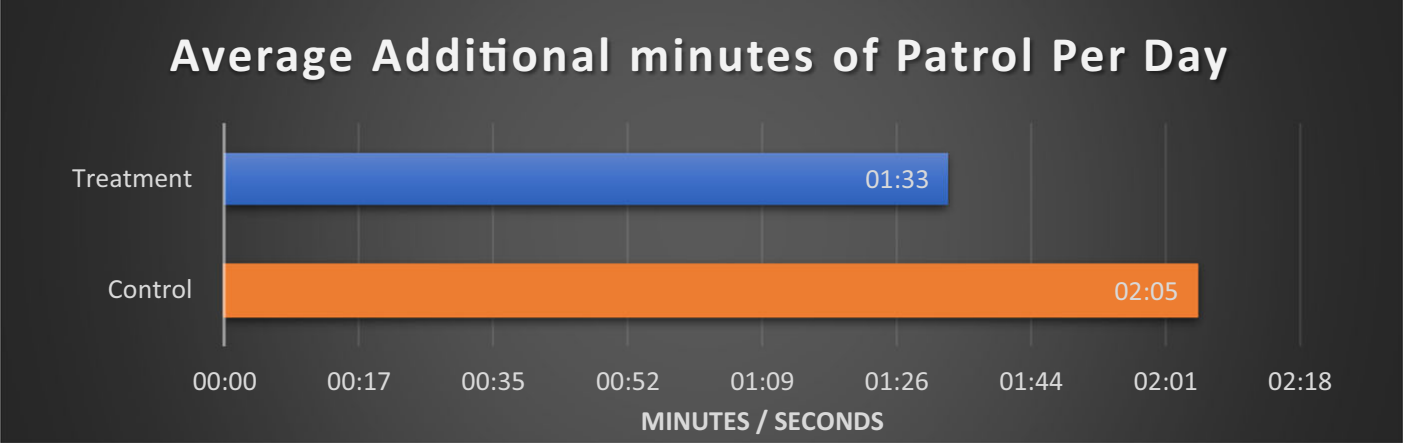
Average additional minutes of patrol presence per day from other sources

As Fig. 3 shows, on control days, each harm spot received an average of 2 min and 5 s from officers either responding to emergency calls or on patrol. With control treatment days on average receiving only 32 s more than patrol treatment days, this finding provides strength in the validity of the treatments influence on the findings.

In addition to these two ways of collecting data (trackers and telematics) to benchmark against the technology being utilised, all officers returned a manual update via email to the dedicated email account providing their entry and exit times to the harm spot, vehicle fleet number (to be discounted against telematics), and other narrative information such as arrests, incidents that occurred and intelligence obtained and nature of the treatment actually delivered. This was descriptive in the words of the patrolling officer to gauge the public response and activity occurring within the harm spot. From an implementation outlook, this was a mechanism that focussed the officers on the core areas they had been asked to deliver in the harm spot and to ensure they carried it out to allow for a worthwhile return.

It must be noted that the use of an external GPS system for the tracking system (handheld device) is a bespoke product utilised for this study with no current emphasis in force on the use of GPS in tracking foot patrol on a day to day basis. This includes the internal GPS system (telematics) which in force was commissioned and funds obtained through the Home Office Innovation Fund with the focus on protecting officers against unfounded complaints, improving fleet management, improving operational deployment, and reducing demand on officers (the system removes the need for log books and mileage returns).

### Compliance

Treatment days totalling 720 were assigned during the 90 days to receive a 15–20-min foot patrol intervention. In total, there were 708 visits with just 12 uncompleted, which provides an overall compliance of 98.33% during the study period. From the officer submissions, narratives to support non-attendance were collated to understand reasons for non-attendance, most of which seemed unavoidable.

#### Findings

These findings present outcomes from a 90-day randomised crossover trial in Southend-on-Sea conducted between 20th July 2020 and 17th October 2020, in which 720 treatment days totalling 220 h of foot patrol compared against 1080 control days were analysed. In these 220 h, foot patrol prevented an estimated 4741 days of recommended imprisonment under English sentencing guidelines, or almost 13 years in prison, for all of the street-visible offences tracked in the experiment.

 Three comparative measures were used to calculate the differences between treatment and control days: 1) sums and means of crime harm, and 2) of crime counts, as well as 3) the use of crime counts and crime harm totals *with outliers removed*. When analysing such police interventions as hot spots to aid comparison of the groups (treatment and control), the removal of outliers may provide a more typical measure of differences in offending between treatment groups (Table 2). This study’s aim was to intervene with foot patrol in harm spots that were generated from the highest harm violent offences. Thus both harm and harm with outliers are analysed to understand the impact the study has had.

**Table 2:** Available sample sizes on successive days of sequential assignment to the same condition—treatment or control

	Control			Treatment		
	N	Percent	Cum. percent	N	Percent	Cum. percent
1st day	435	40.2%	40.2%	433	60.2%	60.2%
2nd day	251	23.2%	63.5%	165	22.9%	83.2%
3rd day	157	14.5%	78.0%	66	9.2%	92.4%
4th day	91	8.4%	86.4%	27	3.8%	96.1%
5th day	53	4.9%	91.3%	18	2.4%	98.5%
6th day	34	3.1%	94.4%	7	1.0%	99.4%
7th day	27	2.5%	96.9%	3	0.4%	99.9%
8th day	13	1.2%	98.1%	1	0.1%	100.0%
9th day	10	0.9%	99.0%			
10th day	6	0.6%	99.5%			
11th day	2	0.2%	99.7%			
12th day	2	0.2%	99.9%			
13th day	1	0.1%	100.0%			
Totals	1080			720		

Table 3 shows that the mean number of community violence crimes (GBH, assault, robbery, and drug dealing) counted on patrol days was 73% lower on days with patrol than on days without patrols. This translates into a Crime Harm CHI difference of a mean of CHI = 1.07 per treatment day in the hot spots, compared to CHI = 9.3 in the same hot spots on control days. The difference is an 88.5% relative reduction in crime harm from the *community violence* offences, the reduction of which was the focus of the funding provided for the experiment.

**Table 3:** Community violence on treatment and control days

Community violence					
	Control	Treatment	Diff. %	*p*	Effect size^a^
Sample size	1080	720			
*Community violence*					
Sum of crime harm	10,045	770	−92.33%	N/A	N/A
Mean of crime harm per day	9.3	1.07	−88.49%	<0.01**	0.81
Sum of crime harm (outliers removed)	4205	770	−92.9%	N/A	N/A
Mean of crime harm (outliers removed)	3.89	1.07	−72.49%	0.01**	0.67
Sum of crime count	34	6	−81.1%	N/A	N/A
Mean of crime count	0.03	0.008	−73.33%	<0.001**	1.6

^a^Cohen’s *d*

^b^Excludes any offences where the crime-harm value exceeds 3 years (1096 days) of recommended incarceration

*p < 0.05, **p < 0.01, ***p < 0.001

### All street visible crime

As Table 4 shows, for all street-visible crime, the mean count of all street-visible crimes per day was 31% lower on treatment days (at 0.09) than on control days (at 0.13). This count difference masked an even larger difference in CHI harm, in which 35.6% lower harm was reported on patrol days (mean CHI = 7.94 days) than on control days (mean CHI = 12.33 days). (The foregoing summary relies on all cases, with no outliers removed.)

**Table 4:** Street visible violence on treatment and control days

Street visible crimes					
	Control	Treatment	Diff. %	*p*	Effect Size^a^
Sample size	1080	720			
*Street visible crimes*					
Sum of crime harm	13,316	5717	−57.1%	N/A	N/A
Mean of crime harm	12.33	7.94	−35.6%	0.11	0.18
Sum of crime harm (outliers removed)	5651	1702	−69.88%	N/A	N/A
Mean of crime harm (outliers removed)	5.23	2.36	−54.88%	0.03*	0.26
Sum of crime count	139	63	−54.86%	N/A	N/A
Mean of crime count	0.13	0.09	−31%	<0.001***	0.66

^a^Cohen’s *d*

^b^Excludes any offences where the crime-harm value exceeds 3 years (1096 days) of recommended incarceration

*p < 0.05, **p < 0.01, ***p < 0.001

Table 4 shows the most comprehensive evidence about the impact of the foot patrol on all types of crime and crime harm that are theoretically deterrable by police presence on public streets. It is the appropriate basis for calculating the cost-effectiveness of the 220 h of patrol delivered in the 90-day test. This calculation requires an adjustment for the different numbers of days in the treatment and control conditions. That adjustment can be done as follows.

#### Adjustment for Unequal Days in Treatment and Control

Taking the finding of a mean level of 7.94 days of harm from street-visible crime in the patrol status, we can subtract that value from the mean of 12.33 CHI days of harm on days in the control group status. The result shows that on each patrol day, a mean of (12.33–7.94=) 4.39 days of CHI-measured harm was saved on each patrol place-day, with 15–20 min of patrol. Since the experiment actually assigned the patrols on 720 treatment place-days, the total CHI days saved was 4.39 per place-day X 720 = 3161—the equivalent of 8.66 years in prison.

## Discussion

This study was led by a “pracademic,” a category of police professionals that may be the key drivers of implementation and compliance outcomes. As Barnes et al. (2020) summarised, “experiments can be conducted with a relatively small team of dedicated police pracademic researchers on patrol”, the exact style in which this study was built and delivered. The pracademic approach balances the meticulous methodological standards rightly expected by science with the more real-world considerations of the practitioners at the front line. This approach also balances the power base observed by the practitioners undertaking the intervention who are more accustomed to formal power in role. Kirch (2007) states that “Culture trumps strategy”, which is why this study’s findings support the move to a more top-heavy and integrated approach to directing implementation in policing. This study has shown that hot spots policing can be achieved through implementation and control from police practitioners with the prerequisite knowledge and drive. This finding echoes those described by De Brito (2016) who found that “The primary mechanism seems to be the human element: differing management styles and capabilities”. Everything observed shows that the consistent delivery of the intervention by a dedicated niche team is the reason for the successful implementation of this experiment.

This study has highlighted that violent crime can be deterred through the use of visible policing, a beacon of guardianship that generated the necessary influence on offenders without direct and traditional contact with them. As demonstrated in the decay of the treatment, its impact was so deep that it drove reductions not only within the study’s key driver, violence, but all street visible crime.

The findings of the study were achieved with a modest allocation of £22,750 of Home Office Surge funding from an allocation of £1,160,000 to Essex Police in the year 2020/21. The initial findings were reported to the Home Office and Policing Minister in December 2020, resulting in Home Office analysis of the data and formal discussions on implementation. The subsequent dialogue and support this research provided has since driven the Violence Reduction Unit Funding Application Guidance (2021a) stipulating that 85% of allocated funding must be utilised to target hot spots using methods proven to deal with violent crime at micro-locations. The activity, methodology, and implementation have all been draw from this Randomised Control Trial (Operation Ark) and published via the Home Office to all forces within the Grip Funding: Hotspots Framework (2021).

A key point is that this study was all delivered using overtime. This dealt with a number of factors that would normally affect such interventions, such as the ability to be shown as additional officers undertaking a bespoke patrol and unavailable for redeployment by the Force Communication Room or Gold Commander. The use of Home Office funding and the requirement for quarterly returns provided the final additional layer of protection in the delivery.

## Conclusion

The experiment has demonstrated significant reductions in crime harm and crime count for community violence, and the possibility to replicate the patrol strategy in other towns and cities.

The study’s findings do tend to support one significant observation. The findings on compliance levels of 98.33% were a major outcome. The protection of the officers from re-deployment in a policing environment that still reacts to demand by all callers ensured that each day, all 8 harm spots allocated could be attended. The last element, and just as significant, is that the officers undertaking the patrol undertook the task knowing they were free from additions to their workloads through incident or crime attendance. These elements weighing on the minds of officers, coupled with the overwhelming cost benefit that dedicated officer patrols provided, lead us to recommend that for a successful and continuous benefit, forces should seek to implement dedicated (specialised) “crime suppression patrol” officers.

The findings of this experiment also provide evidence of strong implementation that further studies can replicate. From initial planning to pre-implementation, there was significant investment focussed on the administration of the experiment and the knowledge briefings delivered to those participating. Each officer was subject to an evidence-based policing input and demonstration of the tracking that would be undertaken. This coupled with deployment packs and daily feedback sought to provide the inclusivity that they were part of the study and subsequent findings. It is by such details that implementation can succeed, and serious violent crimes can be prevented.
